# LncRNA PART1 promotes malignant biological behaviours associated with head and neck cancer cells via synergistic action with FUT6

**DOI:** 10.1186/s12935-024-03372-8

**Published:** 2024-05-28

**Authors:** Yanheng Yao, Yuxin Zhang, Jiyuan Shi, Xiling Xu, Yunran Gao, Suwen Bai, Qin Hu, Jing Wu, Juan Du

**Affiliations:** 1 School of Basic Medical Sciences, Anhui Medical University, 81 Meishan Road, Hefei, 230032 Anhui China; 2 The Second Affiliated Hospital, School of Medicine, Shenzhen & Longgang District People’s Hospital of Shenzhen Guangdong, The Chinese University of Hong Kong, Shenzhen, 518172 China; 3 Ciechanover Institute of Precision and Regenerative Medicine, School of Medicine, The Chinese University of Hong Kong, Shenzhen, 518172 Guangdong China; 4 The First Affiliated Hospital of Anhui Medical University, 218 JiXi Avenue, Hefei, 230022 Anhui China

**Keywords:** Head and neck cancer, LncRNA PART1, FUT6, Apoptosis, Proliferation

## Abstract

The aim of this study was to determine the role of lncRNA PART1 and downstream FUT6 in tumorigenesis and progression of head and neck cancer (HNC). Bioinformatics analysis and qRT-PCR revealed that lncRNA PART1 was expressed at low levels in HNC patients. The proliferation, apoptosis, migration and flow cytometry results showed that low expression of lncRNA PART1 inhibited apoptosis and promoted HNC cell migration and proliferation. In addition, animal experiments have also shown that low expression of lncRNA PART1 can promote tumor growth. LncRNA PART1 overexpression promoted apoptosis and inhibited HNC cell migration and proliferation. Through bioinformatics analysis, FUT6 was found to be expressed at low levels in HNC and to be correlated with patient survival. Immunohistochemical and qRT-PCR results revealed that FUT6 was underexpressed in tumour tissues and HNC cells. Cell and animal experiments showed that overexpression of FUT6 could inhibit tumour proliferation and migration. Bioinformatics analysis revealed that lncRNA PART1 was positively correlated with FUT6. By qRT-PCR and western blot, we observed that after knockdown of lncRNA PART1, both the mRNA and protein expression levels of FUT6 were reduced. The above results indicated that lncRNA PART1 and FUT6 play an important role in HNC, and that lncRNA PART1 affected the development of tumor by downstream FUT6.

**Supplementary Information:**

The online version contains supplementary material available at 10.1186/s12935-024-03372-8.

## Introduction

Head and neck cancer (HNC), which includes lip, mouth, nose, oropharynx, throat and nasopharyngeal cancer, causes almost 700,000 new cases and 380,000 deaths worldwide every year [1]. In addition, head and neck squamous cell carcinoma (HNSCC), which represents 95% of HNC cases, is a heterogeneous biodiverse and genomic disease originating from the squamous mucosa of the upper digestive tract. Approximately 60% of patients with HNSCC have locally advanced or metastatic disease and low survival [2, 3]. Therefore, the discovery of efficient molecular markers and target proteins related to metastatic proliferation is highly important for early diagnosis and treatment and may lead to possible therapeutic targets for patients with HNC.

Long noncoding RNAs (lncRNAs), which are longer than 200 nt, were initially considered transcriptional noise. However, accumulating evidence suggests that they may play a critical role in various cellular processes ranging from normal development to disease [4]. LncRNA dysregulation leads to malignant biological behaviours such as proliferation, invasion and metastasis to promote tumour progression [5–7]. Moreover, lncRNAs have high tissue specificity and stability and thus have potential as biomarkers and therapeutic targets [8, 9]. The lncRNA prostate androgen regulates transcript 1 (PART1) is androgenically regulated in human prostate cancer cells and may play a tumour suppressor role in prostate cancer [10]. Furthermore, downregulation of lncRNA PART1 can promote apoptosis and restrain the proliferation of prostate cancer cells by regulating the Toll-like receptor pathway [11]. In bladder cancer, lncRNA PART1 is a tumour-inhibiting factor that helps to inhibit tumour proliferation and invasion and promote apoptosis [12]. Among patients with oral squamous cell carcinoma, patients with high lncRNA PART1 expression survived longer than did those with low lncRNA PART1 expression [13]. LncRNA PART1 is poorly expressed in glioma, and poor PART1 expression is negatively correlated with overall patient survival [14]. However, the physiological function of lncRNA PART1 in HNC is unclear.

In other aspects, glycosylation, a universal type of protein modification, is involved in many biological processes. Fucosylation is the most common among the numerous types of glycosylation. Abnormal fucosylation and overexpression of fucosyltransferases (FUTs), together with catalytic glycoprotein substrates, have been reported to affect cancer cell proliferation [15]. Thirteen FUT genes have been identified in the human genome and are closely related to cancer pathogenesis and progression [16]. High expression of FUT6 in colorectal cancer could enhance the migration, proliferation, invasion and angiogenesis of colorectal cancer cells in vivo and thereby promote tumour growth [17]. Previous studies have shown that high FUT6 expression is an independent indicator of poor prognosis in patients with acute myeloid leukaemia (AML) and that FUT6 may be a therapeutic target for AML patients [18]. Low FUT6 expression was found in breast cancers with high expression of miR-106b. Downregulation of miR-106b expression in human breast cancer cells increased the expression of FUT6, which led to a significant decrease in the invasion, migration, and proliferation of cancer cells [19]. These interesting results of FUT6 in tumour cells suggest that FUT6 may play different roles in different cancer types. Although emerging evidence suggests that the FUT family could lead to cancer development and progression, its role in HNC has not been documented.

Therefore, the functions of lncRNA PART1 and FUT6 have been extensively studied in HNC. Here, we showed that lncRNA PART1 was downregulated in HNC. Downregulation of lncRNA PART1 promoted the migration and proliferation and suppressed the apoptosis of HN4 and FaDu cells, and decreased FUT6 mRNA expression in these cells. Moreover, in vivo animal experiments have also proved that downregulation of lncRNA PART1 can promote tumor growth. However, overexpression of lncRNA PART1 can inhibit the migration and proliferation of HN4 and FaDu cells, and promote cell apoptosis. Besides, in vivo and in vitro studies showed that the overexpression of FUT6 constrained the migration and proliferation of HN4 and FaDu cells and promoted their apoptosis. In conclusion, lncRNA PART1 play a significant role in promoting HNC development through FUT6, can serve as predictive biomarkers, and can provide a range of potential targets for the treatment of HNC.

## Materials and methods

### Cell culture

HN4 cells and FaDu cells are human HNC cell line, were purchased from KINDU (Shanghai, China) and Servicebio (WuHan, China),cultured in high glucose DMEM containing penicillin‒streptomycin solution and 10% fetal bovine serum (FBS). The human nasopharyngeal epithelial cell line (NP69 cell) was obtained from Fenghui Biological Research (Changsha, Hunan) and cultured in RPMI 1640 medium containing penicillin‒streptomycin solution and 10% FBS. The cells were cultured in a 37 ℃ humidity-controlled incubator with 5% CO_2_.

### Quantitative real time-polymerase chain reaction (qRT-PCR) analysis

Total RNA was extracted from cell or tissue samples with TRIzol reagent (Invitro-gen, USA). The first strand of complementary DNA (cDNA) of FUT6 was transcribed by plus all-in-one 1st Strand cDNA Synthesis SuperMix (JinAn protein, Shanghai). The SYBR green PCR mixture was detected by a Light Cycler 480 machine (Roche, USA). The first cDNA of lncRNA PART1 was transcribed using the LNRCUTE lncRNA first-strand cDNA Kit (Tiangen, Beijing). PCR amplification was performed following the procedures of 40 cycles of 94 °C for 30 s, 60 °C for 30 s, and 72 °C for 30 s. The SYBR green PCR mixture was detected by a QuantStudio3 ma-chine (ABI, USA). The relative quantitative expression of lncRNA PART1 and FUT6 was calculated using the 2^−∆∆CT^ method. The GAPDH was used as an internal reference.

### Cell transfection

The target plasmid of lentiviral vector pCDH-FUT6-MCS-EF1-COPGFP-T2A-PURO or the control plasmid pCDH-CMV-MCS-EF1-COPGFP-T2A-PURO (purchased from General Biology, Anhui) and the package plasmid pSPAX2 and pMD2.G were mixed according to the ratio of pCDH-FUT6-MCS-EF1-COPGFP-T2A-PURO or pCDH-CMV-MCS-EF1-COPGFP-T2A-PURO: pSPAX2: pMD2.G = 4:3:1. The mixed plasmids were transfected into 293 T cells with Lipofectamine 3000. After 48 h, the supernatant of cells containing lentivirus was collected. The obtained lentivirus and polybrene (Biyuntian, Shanghai) were added to HN4 and FaDu cells. Forty-eight hours later, puromycin was added to obtain FUT6-overexpressing cells. The 293 T cells were inoculated into a 6-well plate, and the constructed lncRNA PAPRT1 plasmid pSLenti-EF1-EGFP-F2A-Puro-WPRE2-CMV-PART1 (purchased from Heyuan Biology, Shanghai) and the package plasmids pSPAX2 and pMD2.G were mixed according to the ratio of pSLenti-EF1-EGFP-F2A-Puro-WPRE2-CMV-PART1:pSPAX2:pMD2.G = 4:3:1. The mixed plasmids were transfected into 293 T cells with Lipofectamine 3000. After 48 h of cell culture, the obtained 293 T cell supernatant and 4 µL Polybrene were simultaneously added to the cell supernatant of HN4 cells and FaDu cells for culture, and the medium was changed after 48 h. Then, the HN4 cells and FaDu cells with stable overexpression of lncRNA PAPRT 1 were screened by adding puromycin (puromycin:culture medium = 2.5 µL:1 mL) every day for a week for follow-up experiments. The expression level of lncRNA PART1 in the total RNA of constructed cells was determined by qRT-PCR. Biomics (Shanghai, China) chemically synthesized two small interfering RNAs targeting lncRNA PART1. Specific knockdown of lncRNA PART1 was achieved by transfection of lncRNA PART1 siRNA (200 nM) into HN4 cells using Lipofectamine 3000 and Opti-MEM (Invitrogen; Thermo Fisher Scientific). The expression levels of lncRNA PART1 in the total RNA of the transfected cells were determined by qRT-PCR. All experiments were performed 24 h after transfection.

### Cell proliferation assay

HN4 and FaDu cells were cultured in 96-well plates at a density of 2.0 × 10^5^/cm^2^ in a 37 ℃, 5% CO_2_ humidity-controlled incubator for 24 h. Cell viability was assessed with the cell count kit-8 (CCK-8) (Vazyme, Nanjing, China). A 10% volume of solution was added to the cells and incubated at 37 ℃ for 1 h. The amount of formazan dye generated was directly proportional to the number of viable cells, which was detected at an absorbance wavelength of 450 nm and quantified by an automatic microplate reader.

### Immunohistochemistry

The 5 μm thick tissue sections were dewaxed, dehydrated and subjected to antigen repair. The reagents were added dropwise according to the reagent instructions [ZSGB-BIO, Beijing, Universal SP Kit (Mouse/Rabbit Streptomyces Ovalle in biotin assay system, SP-9000)], stained with DAB solution followed by hematoxylin solution, and finally sealed with neutral gum. The tissues were observed turning a brown‒yellow color under the Olympus microscope, and quantitative immunohistochemical analysis was performed with Image-Pro Plus.

### Transferase-mediated dUTP nick-end labeling (TUNEL) assay

The cells were plated on a polylysine-coated slide, and then 4% paraformaldehyde was added to fix the cells for 30 min at 4 ℃. After that, according to the instructions of the reagent (Vazyme, TUNEL FITC Apoptosis Detection Kit, A111-01), the cells were stained with a dye. Subsequently, the TUNEL-positive cells were analyzed by fluorescence inverted microscopy (Olympus, Japan), and the apoptosis rate was calculated by Image-Plus 6 software.

### Flow cytometry

After digesting the cells with trypsin, the cells were washed twice with PBS and centrifuged at 2000 rpm and 4 ℃ for each wash. Then, 70% precooled ethanol was added to the cells and fixed at 4 ℃ overnight. Subsequently, the fixed cells were stained according to the instructions (C1052, Biyuntien, China), and the cell cycle was tested by flow cytometry (BD/BD FACSCanto II, USA). Data on cell fluorescence were collected to obtain the formation of a univariate karyotype histogram around the fluorescence area signal, and the percentage of nuclei in G1, S and G2/M phases were analyzed by FlowJo software.

### Migration assay

Cells were seeded on 6-well plates until the cells in the wells were confluent, and then the monolayer was scratched with the tip of a sterilized pipette. The medium was then changed to medium without fetal bovine serum. Twelve hours later, the migration of cells along the scratched edges was photographed with a microscope. The results were represented as (S0 − St)/S0, where S0 represents the edge area scratched at the beginning, and St represents the edge area scratched after 12 h.

### Xenograft mouse model of HNC tumor growth

Four-week-old male athymic BALB/C nude mice were purchased from Guangdong Yaokang Biotechnology Co.LTD. The FUT6-overexpressing or transfecting si-lncRNA PART1 HN4 cell lines and the control HN4 cell lines were separately mixed with biological matrix adhesive (Corning, USA) at a ratio of 1:1, and then the mixture was subcutaneously injected into the legs of nude mice at a concentration of 5 × 10^6^ M. Tumor growth was recorded every three days. Four weeks later, the nude mice were sacrificed under isoflurane anesthesia. After size and weight measurements, tumors and organs of nude mice were collected and embedded for further study.

### Statistical analysis

Statistical analysis with t tests was performed using GraphPad Prism V.8 software (GraphPad Software, San Diego, California) to calculate the mean ± SEM of the indicated number of samples. A two-sided value of *p* < 0.05 was considered statistically significant.

## Results

### Low expression level of lncRNA PART1 in head and neck cancer

**Fig. 1:**
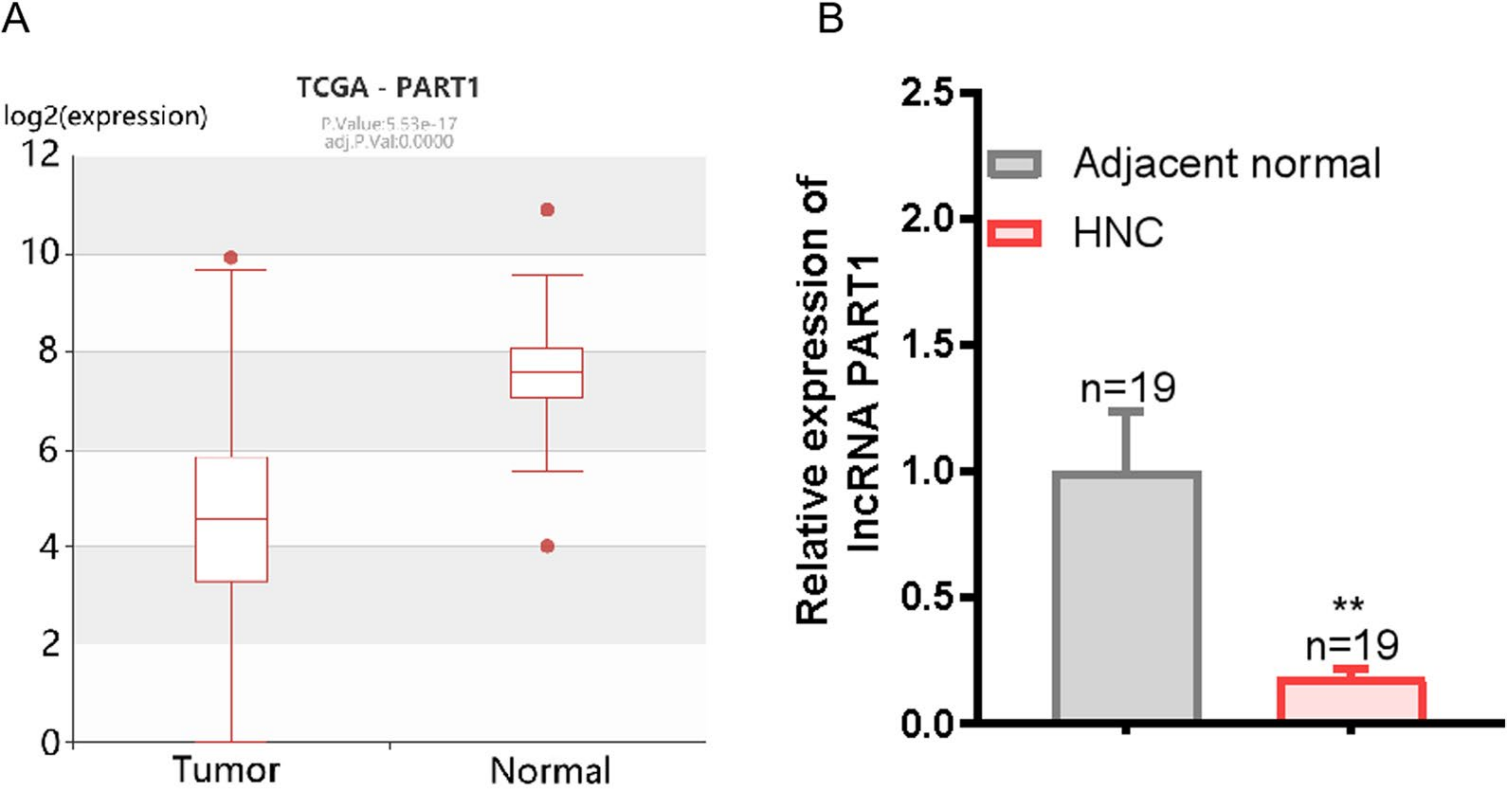
Low expression of lncRNA PART1 in head and neck cancer. **A** Expression of lncRNA PART1 in HNC data from the HNCDB (http://hncdb.canceromics.org/). **B** The relative lncRNA PART1 expression levels were detected via qRT-PCR in HNC tissues (n = 19) and adjacent normal tissues (n = 19). The values represent the means ± SEMs. **p < 0.01, compared with adjacent normal tissues. HNC, head and neck cancer

The genetic information of HNC tissues and their corresponding adjacent normal tissue specimens in the HNC database (HNCDB) were analysed, showing that the expression level of lncRNA PART1 in HNC tissues was markedly lower than that in their normal counterparts (Fig. 1A). Furthermore, we performed qRT-PCR analysis of tumour tissues and adjacent normal tissues from nineteen HNC patients to determine the expression levels of lncRNA PART1 (Fig. 1B). These results showed that lncRNA PART1 plays an important role in HNC.

### Low lncRNA PART1 expression promote malignant behavior in HNC cells

**Fig. 2:**
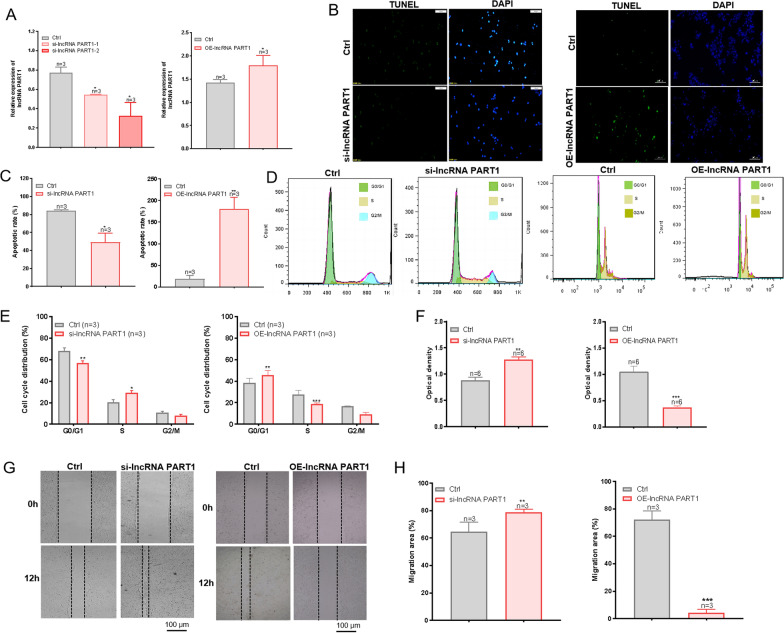
The role of lncRNA PART1 expression in HN4 cells. **A** The expression level of lncRNA PART1 was detected by qRT-PCR after knockdown and overexpression of lncRNA PART1 (n = 3). **B** and **C** A TUNEL assay was used to detect the percentage of apoptotic HN4 cells after knockdown and overexpression of lncRNA PART1 (n = 3). Scale bar = 100 μm. **D** and **E** Representative cell cycle images and summary data on the effects of knockdown and overexpression of lncRNA PART1 on cell cycle (n = 3). **F** A CCK-8 assay was used to detect the effect of knockdown and overexpression of lncRNA PART1 on HN4 cell proliferation (n = 6). **G** and **H** Representative images showing migration and the ratios of cell migration of HN4 cells with knockdown and overexpression of lncRNA PART1 (n = 3). Scale bar = 100 μm.We performed flow cytometry, CCK-8 and scratch experiments at 24 h after transfection of si-lncRNA PART1. The values represent the means ± SEMs. *P < 0.05, **P < 0.01, ***P < 0.001,compared with the control group. si-lncRNA PART1, siRNA-lncRNA PART1. OE-lncRNA PART1,overexpression-lncRNA PART1. Ctrl, control group

We investigated the functional role of lncRNA PART1 in HNC cells by knocking down and overexpressing lncRNA PART1 expression levels in tumor cells. As shown in Fig. 2A, lncRNA PART1 was successfully significantly down-regulated and overexpressed in HNC cells compared to control cells. TUNEL was used to observe the effect of lncRNA PART1 expression on apoptosis. When lncRNA PART1 was knocked down, the apoptosis of HN4 cells was significantly reduced. When lncRNA PART1 was overexpressed, the apoptosis of HN4 cells was significantly increased (Fig. 2B and C). Cell cycle distribution was detected by flow cytometry. As expected, after transfection of si-lncRNA PART1, the proportion of cells in G0/G1 phase decreased significantly, while the proportion of cells in S phase increased significantly. And the proportion of cells in G0/G1 phase increased significantly when lncRNA PART1 was overexpressed. The proportion of cells in the S phase decreased significantly (Fig. 2D and E). The proliferation of HN4 and FaDu cells was detected by CCK-8. The results showed that when lncRNA PART1 was knocked down in HNC cells, the cell proliferation was significantly greater than that in the control group, while the result was opposite when lncRNA PART1 was overexpressed (Fig. 2F and S1A–1B). In addition, scratch experiment results showed that cell migration in the group with low lncRNA PART1 expression was significantly greater than that in the control group, while cell migration in the group with overexpression of lncRNA PART1 was less than that in the control group (Fig. 2G and H). Moreover, in order to determine the effects of lncRNA PART1 on tumor cell proliferation, apoptosis and migration, we also conducted validation experiments in another type of HNC cells, namely FaDu cells. In si-lncRNA PART1 group, the expressions of E-cadherin and BAX were down-regulated, while the expressions of N-cadherin, Vimentin and PCNA were elevated, suggesting that the migration and proliferation of FaDu cells were promoted after lncRNA PART1 down-regulation (Fig. S1C). In lncRNA PART1 overexpression group, the expressions of E-cadherin and BAX were up-regulated, while the expressions of N-cadherin, Vimentin and PCNA were down, suggesting that the migration and proliferation of FaDu cells were inhibited after lncRNA PART1 overexpression (Fig. S1D). These results indicated that the expression level of lncRNA PART1 is closely related to the malignant biological behaviors of HNC cells, such as proliferation, migration, and apoptosis. Therefore, the expression level of lncRNA PART1 may play a crucial role in HNC progression.

### High expression of FUT6 and its predicted relationship with lncRNA PART1 in HNC

**Fig. 3:**
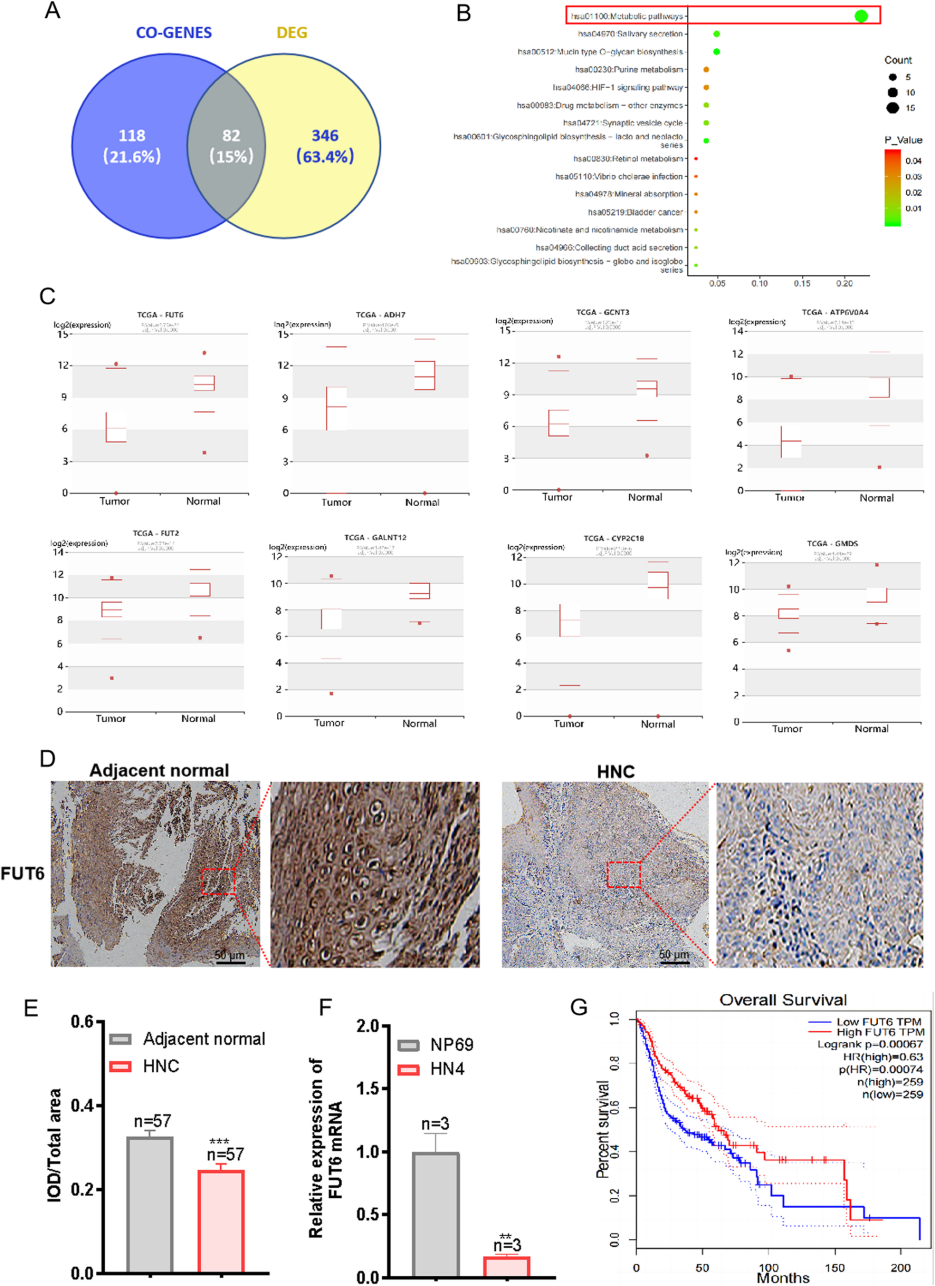
FUT6 expression in HNC and its predicted relationship with lncRNA PART1. **A** Venn diagram showing the intersection of the predicted lncRNA PART1 correlation genes and HNC differential genes. **B** Kyoto Encyclopedia of Genes and Genomes (KEGG) pathway analysis. The top 15 KEGG pathway terms enriched in the intersection of lncRNA PART1-correlated genes and HNC-related DEGs. **C** Eight genes in the TCGA database were expressed at low levels in patients with HNC. **D** FUT6 protein expression in HNC tissues (n = 57) and adjacent normal tissues (n = 57) analysed by immunohistochemistry. The expression of FUT6 was significantly lower in HNC tissues. Scale bar: 50 μm. The magnification is 10×. **E** Quantification of FUT6 protein expression levels in HNC tissues and adjacent normal tissue. **F** The mRNA level of FUT6 in NP69 and HN4 cells was measured by qRT-PCR (n = 3). **G** Kaplan‒Meier survival plot of FUT6 related to OS. Downregulated samples are in blue, while upregulated samples are in red. Survival months are shown along the x-axis. Overall survival rates are shown along the y-axis. The values represent the means ± SEMs. **P < 0.01, ***P < 0.001, compared with the control group. HNC, head and neck cancer

Coexpressed genes (CO-GENES) of lncRNA PART1 and the differentially expressed genes (DEGs) between HNC tissues and the corresponding adjacent normal tissues in the Human Cancer Metastasis Database (HCMDB) were further studied. As shown in Fig. 3A, 82 genes were found at the intersection of the CO-GENES and DEG sets. The KEGG signalling pathways associated with these 82 DEGs were analysed, and the first signalling pathway with P < 0.01 was selected (Fig. 3B). There were 13 genes in this signalling pathway in the HCMDB. The results showed that 8 genes (FUT6, FUT2, ADH7, ATP6V0A4, CYP2C18, GALNT12, GCNT3, and GMDS) had significantly lower expression in patients with HNC (Fig. 3C). Furthermore, the expression levels of FUT6 in adjacent normal tissues and HNC tissues were compared via immunohistochemistry. The results indicated that the expression level of FUT6 was obviously lower in HNC tissues than in adjacent normal tissues (Fig. 3D and E). The mRNA levels of FUT6 in HN4 and NP69 cells were measured via qRT-PCR, the mRNA expression levels of FUT6 were clearly lower in the HN4 cells than in the control cells (Fig. 3F). In addition, to investigate the role of these eight genes in the overall survival of patients with HNC, the relevant data in the HCMDB were further analysed. The Kaplan‒Meier survival curve analysis revealed that the FUT6 level was strongly correlated with overall survival in HNC patients. The lower the expression level of FUT6 was in patients with HNC, the lower the overall survival rate was (Fig. 3G). The differential expression level of FUT6 suggested that FUT6 may play a vital role in the occurrence and development of HNC.

### LncRNA PART1 promotes malignant biological behavior of HNC cells by regulating FUT6 expression

**Fig. 4:**
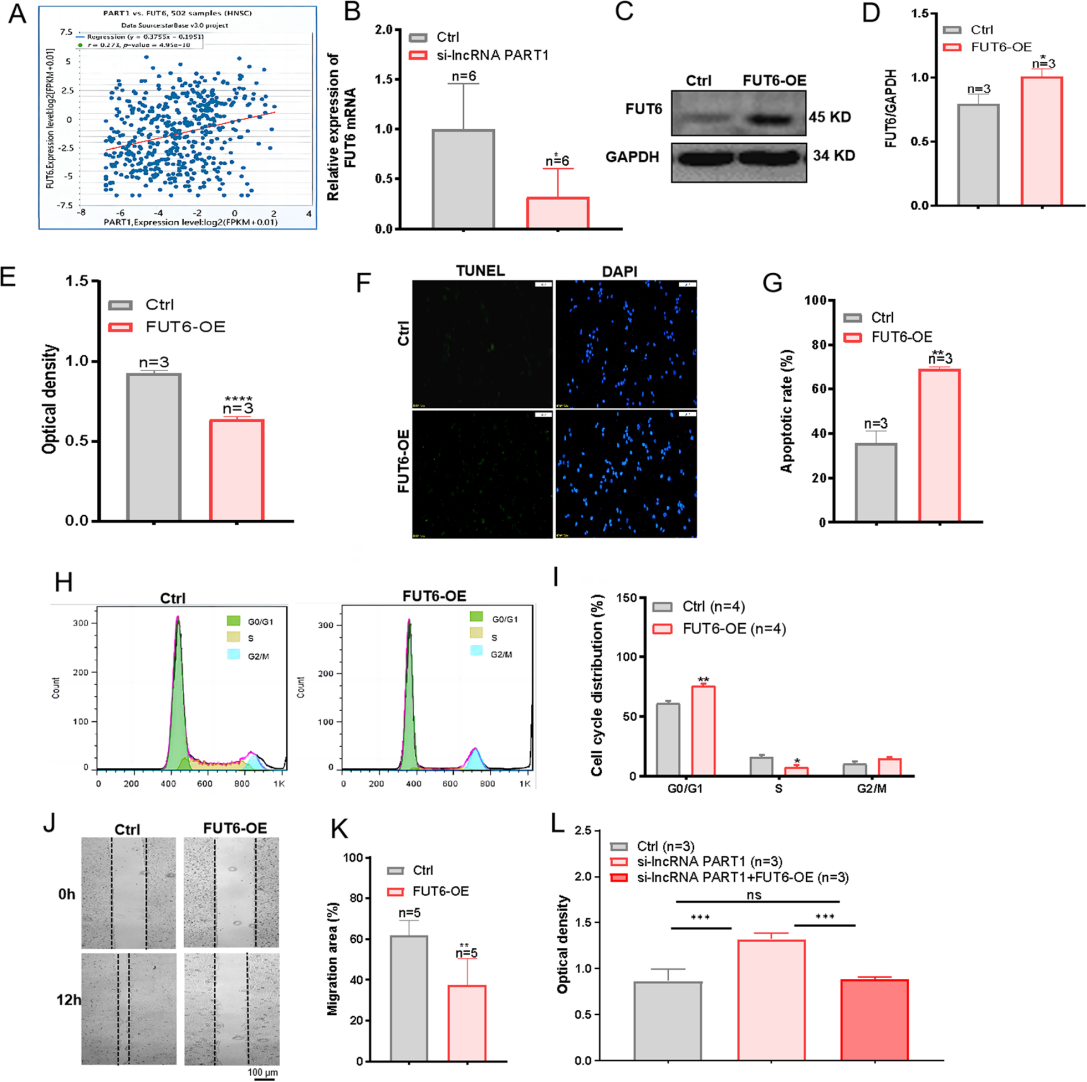
The relationship of lncRNA PART1 with FUT6 and the role of FUT6 in HN4 cells. **A** The correlation between lncRNA PART1 and FUT6 was identified and analysed using starBase V3.0. **B** The mRNA level of FUT6 in HN4 cells was measured by qRT-PCR after si-lncRNA PART1 transfection (n = 6). **C** and **D** Western blot analyzed FUT6 expression after overexpression of FUT6 in HN4 cells. **E** A CCK-8 assay was used to detect the effect of FUT6 overexpression on HN4 cell proliferation (n = 3). HN4 cells were attached to the wall 24 h later and CCK-8 was detected. **F** and **G** A TUNEL assay was used to detect the percentage of apoptotic HN4 cells overexpressing FUT6 (n = 3). Scale bar = 100 µm. **H** and **I** Representative cell cycle images and summary data showing that FUT6 overexpression inhibits proliferation and decreases the number of cells in the S phase (n = 4). **J** and **K** Representative images showing the migration of FUT6-overexpressing HN4 cells and the ratios of cell migration (n = 5). **L** A CCK-8 assay was used to detect upstream and downstream relationship between lncRNA PART1 and FUT6 in HN4 cells. GAPDH was used as a loading control. The values represent the means ± SEMs. *P < 0.05, **P < 0.01, ***P < 0.001, ****P < 0.0001, compared with the control group. FUT6-OE, FUT6 overexpression. Ctrl, control group

The coexpression of FUT6 and lncRNA PART1 was studied based on the starBase V3.0 database, and FUT6 was found to be positively correlated with lncRNA PART1 (Fig. 4A). si-lncRNA PART1-2 was selected for qRT-PCR analysis to detect the mRNA level of FUT6 after lncRNA PART1 knockdown. The mRNA level of FUT6 was obviously lower in the si-lncRNA PART1 group than in the control group (Fig. 4B). Furthermore, western blot results showed that the expression level of FUT6 protein decreased after lncRNA PART1 knockdown (Fig. S2A–B). Therefore, we thought that lncRNA PART1 plays a role in cells by influencing downstream FUT6. In order to investigate the effect of downstream FUT6 on malignant biological behavior of tumor cells, we then constructed two cell lines HN4 and FaDu that overexpressed FUT6 (Fig. 4C–D and S3A). The CCK-8 assay indicated that the proliferation of FUT6-OE HN4 and FaDu cells significantly decreased (Fig. 4E and S3B). The effect of FUT6 overexpression on apoptosis was evaluated by TUNEL assay. Overexpression of FUT6 can significantly increase the apoptosis of HN4 cells (Fig. 4F and G). The cell cycle was further studied by flow cytometry. As shown in Fig. 4H and I, the proportion of cells in G0/G1 phase obviously increased and that of those in S phase significantly decreased in FUT6-OE HN4 cells in comparison with the control group, indicating the diminished proliferation ability of HN4 cells overexpressing FUT6. Moreover, the scratch wound healing assay suggested that, compared with that of the control group, the migration ability of FUT6-OE HN4 cells was significantly lower (Fig. 4J and K). In addition, we also detected proteins related to proliferation, migration and apoptosis in FUT6-OE FaDu cells. In the FUT6-OE group, the expression of N-cadherin, Vimentin, and PCNA was down-regulated, and the expression of E-cadherin and BAX was increased, suggesting that the migration and proliferation of FaDu cells were inhibited after overexpressing FUT6 (Fig. S4A). FUT6 overexpression in HN4 and FaDu cells promoted their apoptosis and reduced their proliferation and migration. Besides, to verify the relationship between lncRNA PART1 and FUT6, CCK-8 assay was performed on untreated HN4 and FaDu cells, HN4 and FaDu cells transfected with si-lncRNA PART1, and HN4 and FaDu cells overexpressed FUT6 after transfection with si-lncRNA PART1, respectively. According to the results in Fig. 4L and S4B, HN4 and FaDu cells transfected with si-lncRNA PART1 showed enhanced proliferation compared with untreated cells. However, the overexpression of FUT6 in HN4 and FaDu cells transfected with si-lncRNA PART1 inhibited the proliferation of these two cells. According to the previous results of CCK-8, cells that overexpress FUT6 have reduced proliferation ability compared with untreated cells. However, when cells overexpress FUT6 on the basis of knocking down lncRNA PART1 in the cells, the results of CCK-8 are basically the same as those of untreated cells. In other words, the effect of decreased FUT6 expression caused by lncRAN PART1 knockdown was offset by FUT6 overexpression. This indicates that lncRNA PART1 can regulate downstream FUT6. Generally, lncRNA PART1 may regulate the proliferation, apoptosis and migration of HN4 and FaDu cells through downstream FUT6.

### The role of FUT6 overexpression and lncRNA PART1 underexpression in HNC cell growth in vivo

**Fig. 5:**
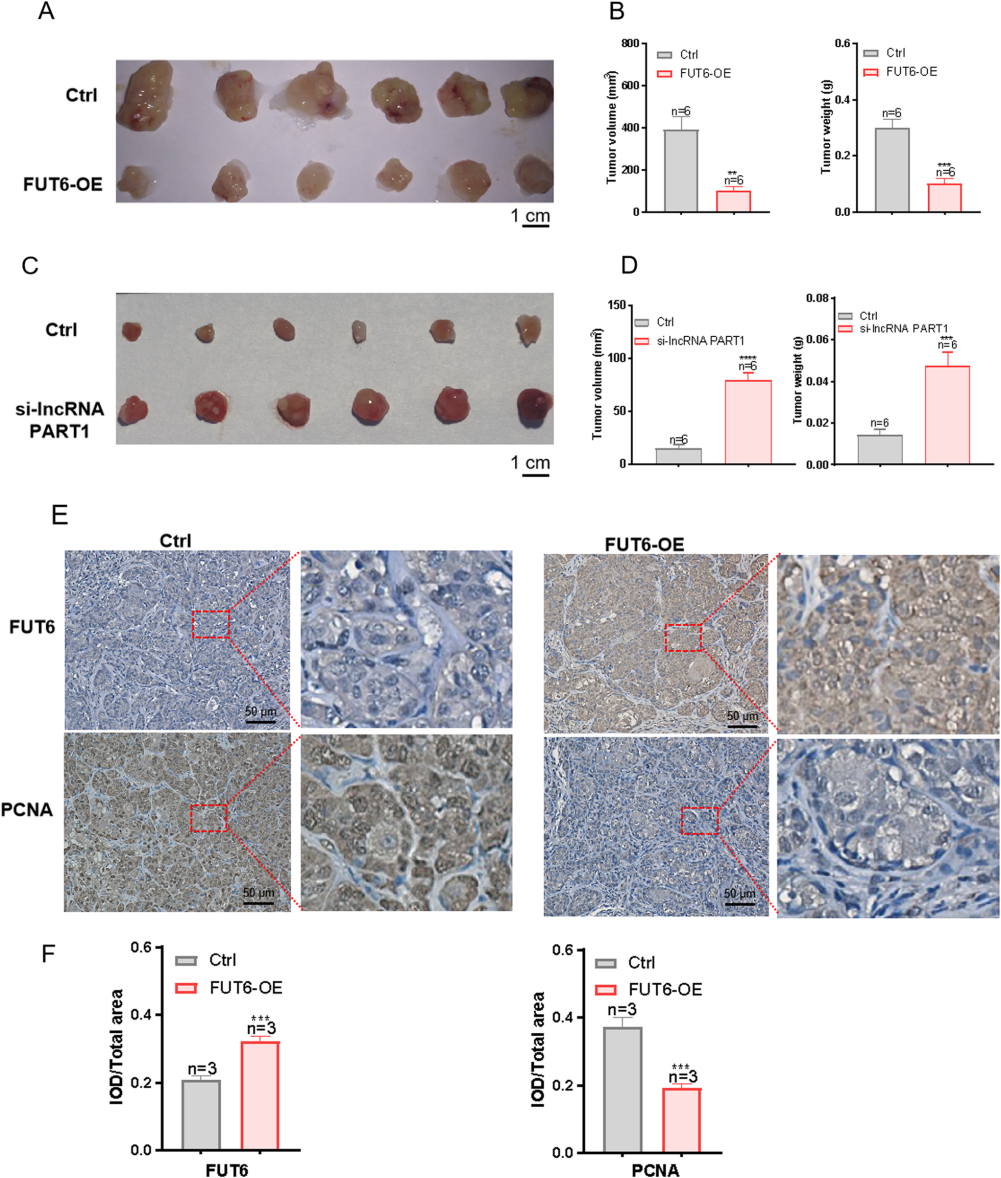
The role of FUT6 overexpression and lncRNA PART1 underexpression in HNC cell growth in vivo*.* **A** Tumours derived from HN4 cells stably overexpressing FUT6 and control HN4 cells were collected (n = 6). **B** Tumour volume and weight were measured in the two groups. **C** Tumours derived from HN4 cells with knockdown of lncRNA PART1 and control HN4 cells were collected (n = 6). **D** Tumour volume and weight were measured in the two groups. **E** FUT6 and PCNA protein expression in FUT6-overexpressing tumour tissues (n = 3) and control tumour tissues (n = 3) analysed via immunohistochemistry. Scale bar: 50 μm. The magnification is 10 × . **F** Quantification of FUT6 and PCNA protein expression levels in FUT6-OE tumour tissues and control tumour tissues. The values represent the means ± SEMs. **P < 0.01, ***P < 0.001, ****P < 0.0001, compared with the control group. FUT6-OE, FUT6 overexpression. si-lncRNA PART1, siRNA-lncRNA PART1. Ctrl, control group

The above in vitro studies indicated that the overexpression of FUT6 might have antitumour effects and underexpression of lncRNA PART1 might promote tumor progression. In vivo experiments on subcutaneous tumour formation in nude mice were performed. FUT6-OE HN4 cells or control HN4 cells transfected with an empty plasmid were subcutaneously injected into the hind limbs of BALB/c-Nu mice. At the same time, HN4 cells transfected with si-lncRNA PART1 or control HN4 cells were injected subcutaneously into the hind limb of BALB/c-Nu mice. Tumour growth in nude mice were observed and recorded every 3 days, and tumours were collected on the 28th day. As shown in Fig. 5A and B, FUT6 overexpression notably impeded tumour growth, as indicated by decreases in tumour weight and volume. As shown in Fig. 5C and D, low expression of lncRNA PART1 can significantly promote tumor growth, manifested as an increase in tumor weight and volume. The expression level of FUT6 in tumours was further studied by immunohistochemical analysis, which showed that the FUT6-overexpressing group had greater FUT6 expression than the control group. Additionally, the expression of the PCNA proliferating protein decreased in the FUT6-overexpressing group in comparison with that in the control group (Fig. 5E and F). These in vivo experimental results showed that overexpression of FUT6 could block tumor growth, while low expression of lncRNA PART1 could promote tumor growth.

## Discussion

LncRNA PART1 and FUT6 play critical roles in tumorigenesis and cancer progression. In this study, we found that lncRNA PART1 was expressed at lower levels in HNC tissues than in adjacent normal tissues. Therefore, we overexpressed and knocked down lncRNA PART1 respectively to explore its role in HNC cells. When lncRNA PART1 was expressed at low level, HN4 and FaDu cells exhibited enhanced proliferation and migration and decreased apoptosis; when lncRNA PART1 was overexpressed, the results were reversed, suggesting that lncRNA PART1 may play an important role in HNC. Interestingly, bioinformatics studies revealed that FUT6 is expressed at lower level in patients with HNC. Moreover, the survival rate was found to be positively correlated with the FUT6 expression level, which indicates that FUT6 gene expression is vital in HNC patients. Immunohistochemistry revealed that FUT6 expression was lower in HNC tissues than in adjacent normal tissues. Furthermore, the results of qRT-PCR demonstrated that the mRNA expression levels of FUT6 were obviously lower in HN4 cells than in NP69 cells. The influence of FUT6 overexpression on the apoptosis, proliferation and migration of HN4 and FaDu cells was further explored. The results of the TUNEL assay showed that FUT6 overexpression notably increased the apoptosis of HN4 cells. Both flow cytometry, CCK-8 assays and western blot showed that the proliferative activity of HN4 and FaDu cells was notably decreased after FUT6 overexpression. In addition, the scratch wound healing assay and western blot suggested that the migration ability of FUT6-overexpressing HN4 and FaDu cells was notably weakened. These results indicate that FUT6 overexpression can promote the apoptosis of HN4 and FaDu cells and inhibit their migration and proliferation. In addition, bioinformatics studies on the coexpression of FUT6 and lncRNA PART1 based on the starBase v3.0 database revealed that FUT6 and lncRNA PART1 were positively correlated. When lncRNA PART1 was knocked down in HN4 cells, the mRNA and protein level of FUT6 decreased, demonstrating that lncRNA PART1 could regulate FUT6. Moreover, the results of CCK-8 also showed that lncRNA PART1 could regulate downstream FUT6. HN4 and FaDu cells transfected with si-lncRNA PART1 showed increased proliferation compared with untransfected cells. However, when FUT6 was overexpressed in HN4 and FaDu cells transfected with si-lncRNA PART1, we found that the overexpression of FUT6 inhibited the proliferation of these two cells. According to previous results from CCK-8, cells that overexpressed FUT6 had a reduced ability to proliferate compared to untransfected cells. Nevertheless, when cells overexpressed FUT6 on the basis of knocking down lncRNA PART1, the results of CCK-8 were essentially the same as those of untreated cells. That is to say, the effect of lncRAN PART1 knockdown on FUT6 expression was offset by FUT6 overexpression. This suggests that lncRNA PART1 plays a role in cells by regulating downstream FUT6. Therefore, the influence of lncRNA PART1 on the proliferation, apoptosis and migration of cancer cells may occur through regulation of the translation and transcription of FUT6.

LncRNAs are involved in multiple physiological processes and play crucial roles in the development of various diseases, including cancer [20]. LncRNA PART1 is prostate androgen regulatory transcript 1, which was first discovered as a new gene that is expressed mainly in the prostate and is regulated by androgens in human prostate cancer cells [10]. It has also been reported that downregulating lncRNA PART1 expression could regulate the Toll-like receptor pathway, which inhibits the proliferation of prostate cancer cells and promotes their apoptosis. Studies on colorectal cancer have shown that the expression level of lncRNA PART1 is increased in cancer tissues and cells and that the proliferation and metastatic capacity of colorectal cancer cells are reduced after downregulation [21]. Our studies showed that lncRNA PART1 was underexpressed in patients with HNC and that its low expression could promote cancer cell migration and proliferation and inhibit cancer cell apoptosis.

On the basis of bioinformatics analysis, one gene from the fucosyltransferase family, FUT6, was found to be significantly associated with overall survival in patients with HNC. Fucosylation is one of the most important types of glycosylation in cancer [22]. FUT6 is a biosynthetic enzyme in the FUT family, which can be used to catalyse the transfer of fucose from guanosine diphosphate β-l-fucose to various sugar substrate receptors on oligosaccharides, glycoproteins and glycophospholipids [23]. In total, 13 FUT genes, which are closely related to pathological processes such as cancer occurrence and tumour progression, have been found in the human genome. For example, FUT4 is involved in the metastasis and proliferation of breast cancer cells and can be used as a potential biomarker for the diagnosis and prognosis of breast cancer [24]. FUT5 and FUT6 are highly expressed in colorectal cancer and promote tumour growth by promoting the proliferation, invasion, migration and angiogenesis of colorectal cancer cells in vivo [17]. Low expression of FUT6 has been found in breast cancers with high expression of miR-106b. Downregulation of miR-106b in human breast cancer cells can increase FUT6 expression, leading to a significant decrease in the migration, invasion, and proliferation of cancer cells [19]. In this study, we found that FUT6 expression was decreased in HNC patients. The lower the expression of FUT6 was, the lower the overall survival rate of patients with HNC was. The migration and proliferation abilities of HN4 and FaDu cells were reduced, and apoptosis was enhanced after FUT6 was overexpressed. Similarly, in vivo experiments demonstrated that the proliferative capacity of tumour cells decreased after FUT6 was overexpressed. Therefore, FUT6 may be an effective target for preventing the development and metastasis of HNC.

Generally, lncRNAs can regulate gene expression within the nucleus at both the epigenetic and transcriptional levels or at the posttranscriptional and translational levels in the cytoplasm [25]. In addition, lncRNAs are reportedly involved in various biological processes, including sponging miRNAs to translate mRNAs, maintaining protein stability, and regulating glucose metabolism and cell signal transduction [26–28]. In tongue squamous cell carcinoma tissues and cell lines, lncRNA PART1 was found to be expressed at low levels, while miR-503-5p was highly expressed. Further studies revealed that lncRNA PART1 could play a tumour suppressive role in tongue squamous cell carcinoma by targeting miR-503-5p [29]. LncRNAs have also been reported to regulate mRNA stability or translation and affect cellular signalling cascades. For example, the lncRNA Staufen-1 was demonstrated to mediate mRNA attenuation by interacting with double-stranded RNA in the 3’UTR of mRNAs [30]. Markus et al. performed an in-depth study of the lncRNA TINCR, a critical lncRNA required for somatic histodifferentiation, through its interaction with a series of differentiated mRNAs [31]. LncRNA-p21 was also reported to selectively inhibit the translation of mRNAs by interacting with JUNB and CTNNB1 mRNA[32]. In our studies, lncRNA PART1 was found to be positively correlated with FUT6. When lncRNA PART1 was knocked down in HN4 and FaDu cells, the mRNA expression levels of FUT6 were reduced, indicating that lncRNA PART1 could regulate the translation and transcription of FUT6. In other words, lncRNA PART1 may affect the proliferation, apoptosis and migration of HNC cells through regulation of downstream FUT6.

### Supplementary Information

Supplementary material 1.

## Data Availability

The datasets generated during and/or analyzed during the current study are available from the corresponding author on reasonable request.
