# Supplementary material for: LncRNA PART1 promotes malignant biological behaviours associated with head and neck cancer cells via synergistic action with FUT6
Source: Cancer Cell Int. 2024 May 28;24:185. doi: 10.1186/s12935-024-03372-8 (PMC11134962; doi:10.1186/s12935-024-03372-8)
Supplement: Supplementary file 1 — Supplementary material 1. [file 12935_2024_3372_MOESM1_ESM.docx]

Supporting Information

**LncRNA PART1 promotes malignant biological behaviours associated with head and neck cancer cells via synergistic action with FUT6**

**Yanheng Yao ^1^****^†^,** **Yuxin Zhang ^1†^,Jiyuan Shi ^1†^, Xiling Xu ^1^,****Yunran Gao** **^1^, Suwen Bai** **^2^, Qin Hu^5^*, Jing Wu ^4^*, Juan Du ^1,2, 3^***

^1^School of Basic Medical Sciences, Anhui Medical University, 81 Meishan Road, Hefei, Anhui 230032, China.

^2^The Second Affiliated Hospital, School of Medicine, The Chinese University of Hong Kong, Shenzhen & Longgang District People's Hospital of Shenzhen Guangdong 518172, China.

^3^Ciechanover Institute of Precision and Regenerative Medicine, School of Medicine, The Chinese University of Hong Kong, Shenzhen, Guangdong 518172, China.

^4^The First Affiliated Hospital of Anhui Medical University, 218 JiXi Avenue, Hefei 230022, Anhui, China.

^5^Kobilka Institute of Innovative Drug Discovery, School of Medicine, The Chinese University of Hong Kong, 2001 Longxiang Boulevard, Longgang District, Shenzhen , Guangdong 518172, China.

Table of Contents

1. Table of DNA sequences S**3**

2. CCK-8 and western blot results of FaDu cells transfected with si-lncRNA PART1 and overexpressed lncRNA PART1 S**4**

3. Western blot analyzed FUT6 expression after transfecting with si-lncRNA PART1 in HN4 and FaDu cells S**5**

4. Results of CCK-8 and western blot of FaDu cells overexpressing FUT6 .S**6**

5.Western blotting of malignant biobehavior-related proteins in FaDu cells overexpressing FUT6 and LncRNA PART1 regulates malignant behavior of FaDu cells through FUT6 by CCK-8 analysis S**7**

**Table S1**. The DNA sequences used in this work.

| Name | Sequences (5'-3') |
| --- | --- |
| LncRNA PART1-forward | CTCTGGAAAGCTGAAAGGGCT |
| LncRNA PART1 -reverse | TGTCCTTTTCCCCTCCGACA |
| FUT6-forward | GCCTTTTAACAAACCCATAGCT |
| FUT6-reverse | GTTGTACATGACCTCTCGGTG |
| GAPDH-forward | GGGGTCATTGATGGCAACAATA |
| GAPDH-reverse | ATGGGGAAGGTGAAGGTC |
| si-lncRNA PART1#1 | CTGTATGAATC GCCA TGAA GACT |
| si-lncRNA PART1#2 | CCGCTAAACTGGACATTTCAAGA |


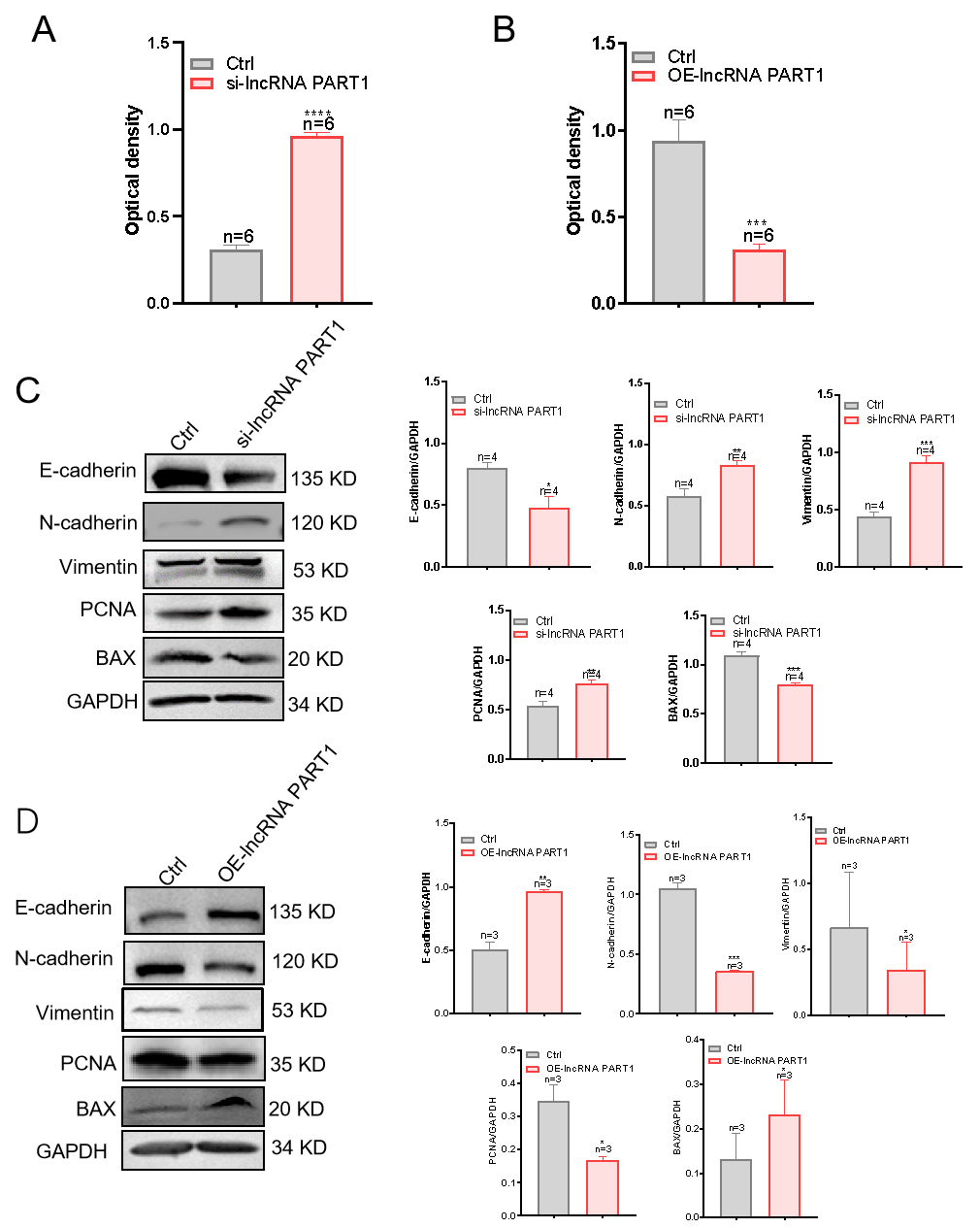


**Figure S1 Role of** **lncRNA PART1 expression in proliferation, migration and apoptosis of FaDu cells. (A)** A CCK-8 assay was used to detect the effect of si-lncRNA PART1 on FaDu cell proliferation. **(B)** A CCK-8 assay was used to detect the effect of overexpression of lncRNA PART1 on FaDu cell proliferation. **(C)** Western blot analyzed E-cadherin, N-cadherin, Vimentin, PCNA and BAX protein in FaDu cells after transfecting with si-lncRNA PART1. **(D)** Western blot analyzed E-cadherin, N-cadherin, Vimentin, PCNA and BAX protein in FaDu cells with overexpression of lncRNA PART1. GAPDH was used as a loading control. The values represent the means ± SEMs. **P*<0.05, ***P*<0.01, ****P*<0.001, compared with the ctrl group. FUT6-OE, FUT6 overexpression. si-lncRNA PART1, siRNA-lncRNA PART1.Ctrl, control group.


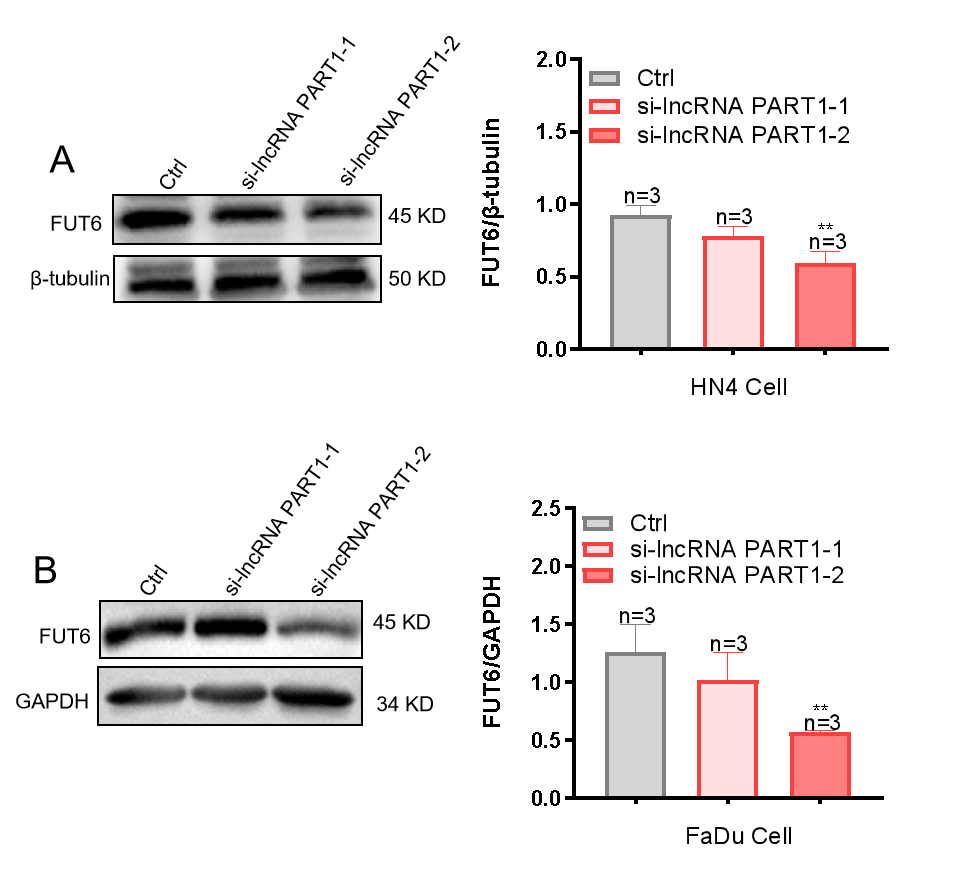


**Figure S2** **LncRNA PART1 regulates FUT6 expression in HNC cells.** **(A)** and **(B)** Western blot analyzed FUT6 expression after transfecting with si-lncRNA PART1 in HN4 and FaDu cells (n=3). β-Tubulin and GAPDH were used as a loading control. The values represent the means ± SEMs. ***P*<0.01, compared with the ctrl group. si-lncRNA PART1, siRNA-lncRNA PART1. Ctrl, control group.


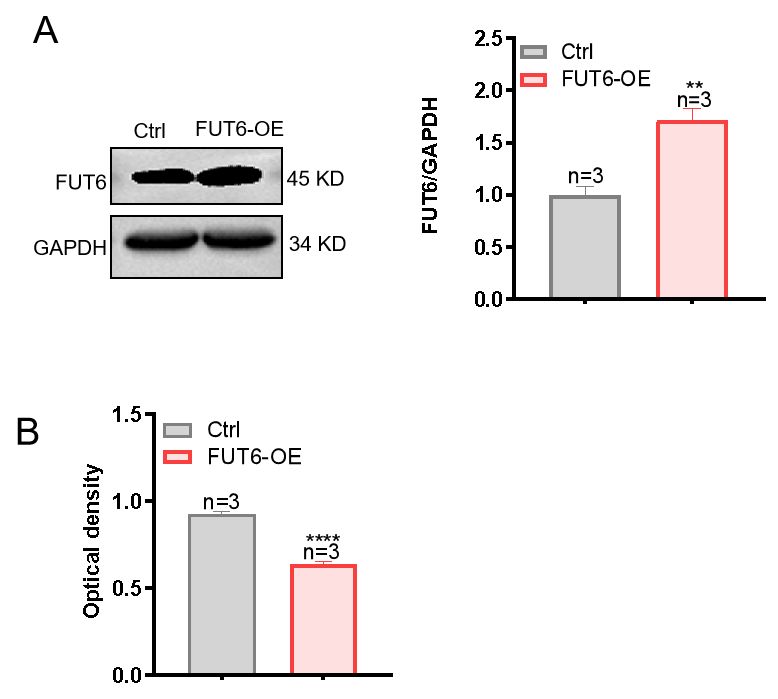


**Figure S3 Construction of FaDu cells overexpressing FUT6 and effect of overexpressing FUT6 on proliferation of FaDu cells.** **(A)** Western blot analyzed FUT6 expression after overexpression of FUT6 in FaDu cells (n=3). **(B)** A CCK-8 assay was used to detect the effect of FUT6 overexpression on FaDu cell proliferation (n=3). GAPDH was used as a loading control. The values represent the means ± SEMs. ***P*<0.01, *****P*<0.0001, compared with the ctrl group. FUT6-OE, FUT6 overexpression. Ctrl, control group. Ctrl represents the cells transfected with the control plasmid.


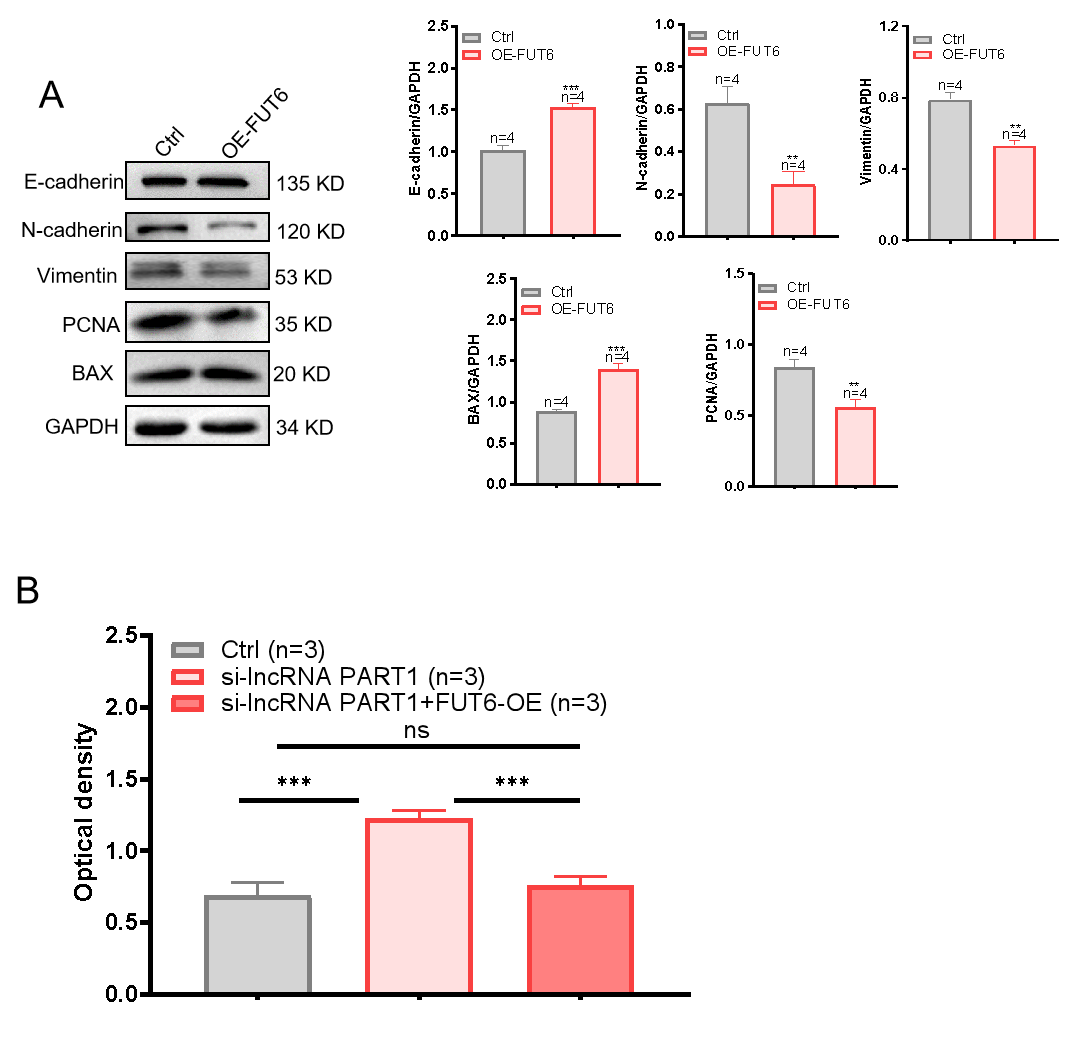


**Figure S4 LncRNA PART1 regulates the malignant behavior through FUT6 in FaDu cells. (A)** Western blot analyzed E-cadherin, N-cadherin, Vimentin, PCNA and BAX protein in FaDu cells over-expressed FUT6. **(B)** A CCK-8 assay was used to detect upstream and downstream relationship between lncRNA PART1 and FUT6 in FaDu cells. GAPDH was used as a loading control. The values represent the means ± SEMs. ***P*<0.01,****P*<0.001, compared with the ctrl group. FUT6-OE, FUT6 overexpression. si-lncRNA PART1, siRNA-lncRNA PART1. Ctrl, control group.
